# Profiles of Peripheral Immune Cells of Uncomplicated COVID-19 Cases with Distinct Viral RNA Shedding Periods

**DOI:** 10.3390/v13030514

**Published:** 2021-03-19

**Authors:** Denise Utami Putri, Cheng-Hui Wang, Po-Chun Tseng, Wen-Sen Lee, Fu-Lun Chen, Han-Pin Kuo, Chih-Hsin Lee, Chiou-Feng Lin

**Affiliations:** 1 Pulmonary Research Center, Wanfang Hospital, Taipei Medical University, Taipei 116, Taiwan; 108336@w.tmu.edu.tw; 2 Department of Laboratory Medicine, Wanfang Hospital, Taipei Medical University, Taipei 116, Taiwan; 93059@w.tmu.edu.tw; 3 School of Medical Laboratory Science and Biotechnology, College of Medical Science and Technology, Taipei Medical University, Taipei 110, Taiwan; 4 Department of Microbiology and Immunology, School of Medicine, College of Medicine, Taipei Medical University, Taipei 110, Taiwan; pctseng@tmu.edu.tw; 5 Core Laboratory of Immune Monitoring, Office of Research and Development, Taipei Medical University, Taipei 110, Taiwan; 6 Divisions of Infectious Diseases, Department of Internal Medicine, Wanfang Hospital, Taipei Medical University, Taipei 116, Taiwan; 89425@w.tmu.edu.tw (W.-S.L.); 96003@w.tmu.edu.tw (F.-L.C.); 7 Divisions of Pulmonary Medicine, Department of Internal Medicine, School of Medicine, College of Medicine, Taipei Medical University, Taipei 110, Taiwan; q8828@tmu.edu.tw; 8 Divisions of Pulmonary Medicine, Department of Internal Medicine, Wanfang Hospital, Taipei Medical University, Taipei 116, Taiwan; 9 Graduate Institute of Medical Sciences, College of Medicine, Taipei Medical University, Taipei 110, Taiwan

**Keywords:** COVID-19, immune profile, viral RNA-shedding, viral RNA clearance

## Abstract

The heterogeneity of immune response to COVID-19 has been reported to correlate with disease severity and prognosis. While so, how the immune response progress along the period of viral RNA-shedding (VRS), which determines the infectiousness of disease, is yet to be elucidated. We aim to exhaustively evaluate the peripheral immune cells to expose the interplay of the immune system in uncomplicated COVID-19 cases with different VRS periods and dynamic changes of the immune cell profile in the prolonged cases. We prospectively recruited four uncomplicated COVID-19 patients and four healthy controls (HCs) and evaluated the immune cell profile throughout the disease course. Peripheral blood mononuclear cells (PBMCs) were collected and submitted to a multi-panel flowcytometric assay. CD19^+^-B cells were upregulated, while CD4, CD8, and NK cells were downregulated in prolonged VRS patients. Additionally, the pro-inflammatory-Th1 population showed downregulation, followed by improvement along the disease course, while the immunoregulatory cells showed upregulation with subsequent decline. COVID-19 patients with longer VRS expressed an immune profile comparable to those with severe disease, although they remained clinically stable. Further studies of immune signature in a larger cohort are warranted.

## 1. Introduction

The coronavirus disease 2019 (COVID-19) is an ongoing disaster causing a catastrophic loss in lives and socioeconomic well-being worldwide. The clinical presentation varies widely from asymptomatic cases, mild respiratory symptoms, and fever to severe organ failure, septic shock, and death. Critical inflammatory response and acute lung injury, in addition to lymphopenia and cytokine release syndrome, have been reported as critical clinical features of the COVID-19 patients, especially among those with comorbidities [1,2,3]. Current policy in several countries involves antibody testing in determining immunity to the SARS-CoV-2 infection, and a study has indicated 90% of severe COVID-19 patients develop IgG antibodies within the first 2 weeks of symptomatic infection, which coincides with the disappearance of the virus [4]. However, a key question concerns antibodies in asymptomatic and mild disease individuals, as the population may present with low virus-binding antibody titers [5,6].

COVID-19 patients present with heterogeneity of immune response [7]. Understanding the actual physiological and immunological processes along the disease course is crucial to identifying and rationalizing effective treatments and policymaking. While discordance between the severity of clinical presentations and the infectiousness has been brought to public attention, little is known about how the immune cells evolved in mild COVID-19 cases with a prolonged SARS-CoV-2 viral RNA shedding (VRS) period. We identified uncomplicated COVID-19 patients with distinct viral RNA conversion time and evaluated the peripheral immune cells to expose the immune system’s interplay. Moreover, the immune cell profile’s dynamic changes in the prolonged cases were also evaluated along the disease course.

## 2. Materials and Methods

### 2.1. Subject Characteristics

Four adult patients admitted to a tertiary medical center with confirmed COVID-19 diagnosis, according to World Health Organization interim guidance and positive real-time reverse polymerase chain reaction (PCR) examination from throat/nasal swab samples, were included in the study. Patients were discharged in the absence of fever or dyspnea for at least 3 days, improvement in both lungs on radiography, if any, and three consecutive nasal-swab samples plus a sputum sample negative for viral RNA obtained at least 24 h apart. Concurrently, four adults (age 22, 45, 52, and 56 years old, one male) with no travel and contact history nor presenting symptoms were recruited as healthy controls (HCs). Subject demographics and clinical information are shown in Table 1.

**Figure 1:**
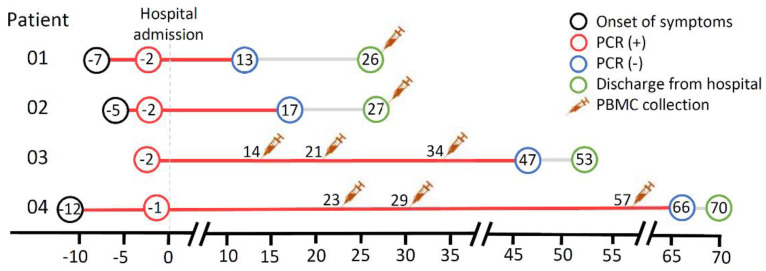
Disease course and peripheral blood mononuclear cell (PBMC) collection timeline of the patients. PCR: polymerase chain reaction assay (from nasopharyngeal swab).

A recent study reported a median time of VRS of 19 days following symptom onset, with the longest being 44 days [8]. Patients 01 and 02 had a total of 20 and 22 days of positive VRS, calculated from the symptom onset to three consecutive negative PCR results, respectively. Patients 03 and 04 had a prolonged positive VRS of 49 and 78 days (Table 1; Figure 1). All patients presented with no symptoms requiring supplemental oxygenation or critical care, consistent with uncomplicated cases.

### 2.2. Isolation of Peripheral Blood Mononuclear Cells (PBMC)

Venous blood was drawn from subjects to collect mononuclear cells from blood buffy coats through separation using SepMate tubes (STEMCELL Technologies, Vancouver, BC, Canada) according to the manufacturer’s instructions. In brief, heparinized blood was diluted two-fold with phosphate-buffered saline, layered on top of Lymphoprep, and centrifuged at 1200× *g*, for 10 min with the brake on. PBMCs were collected by pouring supernatant into a 50 mL polypropylene tube, washed twice with PBS, and counted using a hemacytometer with trypan blue (Lonza, NH, USA) to determine cell viability.

### 2.3. Staining and Flow Cytometry Analysis

PBMCs were submitted to flow cytometry immunofluorescence assay using Attune Nxt Flow Cytometer (Thermo Fisher Scientific, Waltham, MA, USA) according to the manufacturer’s recommendation. After washing with PBS, Fc receptors were initially blocked using Human FcR blocking reagent (Miltenyi Biotec, Bergisch Gladbach, Germany) for 30 min at 4 °C, followed by cell surface labeling by specific primary antibodies. The following antibodies were adapted into multiple panels: CD4, CD8, CD14, CD11c, CD16, CD19, CD25, CD62L, HLA-DR, CD56, CD45RA, CD45RO, CCR3, CCR5, CCR6, CCR10, CXCR3, and CXCR5 (Thermo Fisher Scientific, Waltham, MA, USA). Results were illustrated as the percentage of positive cells or as the ratio of the mean of fluorescence intensity (MFI) from the antibody of interest to the isotype control antibody.

### 2.4. Statistical Analysis

Data from flow cytometry were analyzed using GraphPad Prism (La Jolla, CA, USA). Mean values with standard deviations were presented in the data graphs, and Student’s *t*-test or one-way ANOVA analysis of variance followed by Newman–Keuls’ posthoc test was performed for statistical analysis. Results with *p*-values < 0.05 were considered significant.

## 3. Results

We first scrutinized the frequency of peripheral immune cells from the four patients as a ratio to the mean of HCs. Peripheral blood mononuclear cells (PBMCs) from patients 01 and 02 were collected on the day of discharge, thus in the recovery (negative VRS) state. In contrast, for patients 03 and 04, PBMCs were isolated on days 14 and 23 of hospitalization, respectively, during the active disease (positive VRS) state. For further analysis of the kinetics of immune cell expression throughout the disease course, two consecutive blood drawings following the first were obtained from patients 03 and 04. PBMCs were collected from patient 03 on days 21 and 34, and patient 04 on days 29 and 57, of hospital stay, respectively; all were during the active disease state (Figure 1).

**Figure 2:**
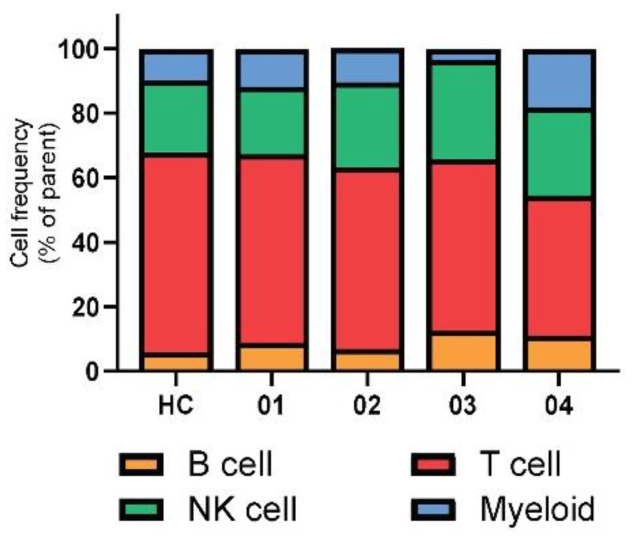
The overall profile of immune cell compartments of healthy controls (HC) (*n* = 4), presented as average, and patients 01–04; data show the percentage of total PBMC obtained in flow-cytometry.

**Figure 3:**
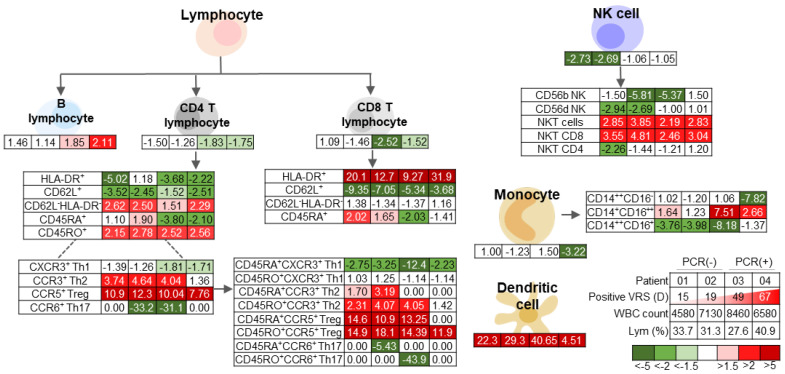
Peripheral immune cell profile of patients 01–04 relative to the mean of HC; number presented as a fold-change ratio. PCR: Polymerase Chain Reaction assay (from nasopharyngeal swab); PBMC: peripheral blood mononuclear cell; VRS: Viral RNA Shedding; d: days; WBC: white blood cell; Lym: lymphocyte.

**Figure 4:**
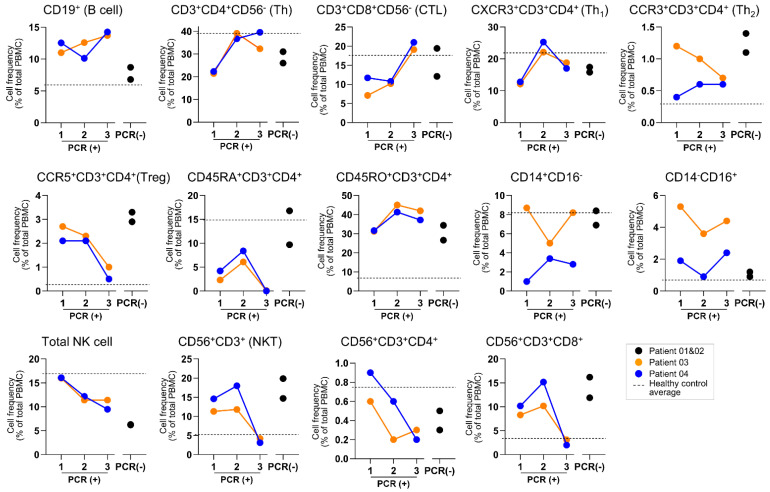
Time-course frequency of peripheral immune cells in prolonged COVID-19 patients expressed in % of total parent cells. PBMCs from patients 01 and 02 (black) were collected on the day of hospital discharge (day 26 and 27, respectively), while PBMCs from patients 03 (orange) and 04 (blue) were collected during active disease state (days 14, 21, 34; and 23, 29, 57, respectively).

An overall profile of the immune cell compartment is presented in Figure 2. We discovered a lower T cell population and a higher B cell in COVID-19 patients than HCs. Patient 03 was remarkably lower, while patient 04 showed a higher myeloid cell compartment than HCs and the shorter VRS cases. To further understand the in-depth expression of immune cell subsets, we applied a multi-parameter staining strategy to differentiate at least 35 distinct immune cell subsets (Figure 3). Although our cohort presented with normal lymphocyte count, CD19^+^-B cells were notably upregulated in both prolonged VRS cases, while CD3^+^CD56^−^CD4^+^ (CD4) cells and CD3^+^CD56^−^CD8^+^ (CD8) cells were downregulated. Along the extended disease course, evident recovery of T lymphocytes was observed, while B lymphocytes remained higher compared to both the shorter VRS patients and HCs (Figure 4). CD62L^+^ cells comprised of both naïve and central memory T cells were downregulated in both CD4 and CD8 populations of all confirmed patients. Further, CD45RA^+^ expressing-CD4 cells showed brief upregulation and downregulation in shorter and prolonged VRS cases compared to HCs, respectively. The effector CD4 cells represented by CD62L^−^HLA-DR^−^ and the memory CD4 cells represented by CD45RO^+^ were prominently upregulated relative to HCs, as expected in the course of infection [9]. However, no noticeable difference was observed in the CD62L^−^HLA-DR^−^CD8 cells.

Th1 subset population, represented by CXCR3^+^CD3^+^CD4^+^ markers, showed unique downregulation on the prolonged cases, followed by improvement along the disease course, to a comparable level with the shorter VRS patients and the HCs. Meanwhile, the anti-inflammatory subset Th2 and Treg, represented by CCR3^+^CD3^+^CD4^+^ and CCR5^+^CD25^+^CD3^+^CD4^+^ markers, respectively, both the naïve and memory phenotypes, were upregulated in our COVID-19 cohort relative to HCs, suggesting immunoregulation and counter-inflammatory mechanisms [10]. In the prolonged cases, Treg population further showed an apparent decline as the disease progressed, to a level comparable with HCs, while the expression remained high in recovered patients with shorter VRS.

Overall, the NK cell population was lower in patients with a shorter VRS period than HCs, which was dominated by circulating the CD56d-NK subtype. In contrast, the frequency of CD8^+^CD56^+^-NKT cells, notably on CD8^+^CD56^+^CD3^+^-NKT CD8 cell population, was higher in the COVID-19 cohort, regardless of their VRS period. Both NK cells and NKT cell populations later decreased along the disease course in patients 03 and 04, while the NKT cell number remained high in patients 01 and 02. 

Compared to HCs, CD11c^+^HLA-DR^+^-dendritic cells (DCs) were upregulated regardless of the VRS period. The total monocyte number was uniquely downregulated on the patient with the most extended VRS period. These monocytes were primarily of the CD14^++^CD16^−^-classical monocyte subsets (data not shown); thus, downregulation on the most extended VRS period patient was apparent in this subset. The frequency remained relatively steady throughout the disease course. CD14^++^CD16^+^ non-classical monocytes were upregulated on both prolonged VRS period patients, while CD14^++^CD16^+^ intermediate monocytes were downregulated unless for the most extended VRS period patient.

## 4. Discussion

Transmitted primarily via respiratory droplets, the viral load of SARS-CoV-2 reaches its peak within 5–6 days of symptom onset [11,12]. Released viral RNA is recognized as a pathogen-associated molecular pattern and triggers a local immune response by recruiting macrophages and monocytes, followed by priming of adaptive T and B cells [3]. Recruitment of immune cells, notably lymphocytes, into the airway may explain the lymphopenia commonly observed from peripheral blood count in patients [13]. Although WBC and lymphocyte counts remained within the normal range in our cohort, patients with prolonged VRS period displayed notably higher and lower frequencies of B and T cells, respectively. Previous studies have similarly reported a decline in CD4 and CD8 cells in acute moderate or severe COVID-19 cases, which improved during the resolution period [7,14,15]. Dissecting further, HLA-DR^+^CD4^+^ cells were downregulated, while HLA-DR^+^CD8^+^ were upregulated, in COVID-19 patients compared to HCs. Earlier studies have also reported a high frequency of CD38^+^HLA-DR^+^CD8^+^ T cells in uncomplicated COVID-19 cases (compared to healthy control) and resolved severe cases (compared to severe persistent cases). However, the observation on CD38+HLA-DR^+^CD4^+^ T cells was contradictory with our current result [16,17]. Co-expression of CD38 and HLA-DR uniquely represents cell activation to viral infection [18], while HLA-DR expression reflects a more general T cell activation. Thus, the discrepancy may not be directly explained between these studies.

Dissecting the Th cell phenotypes, we observed an apparent lower CXCR3^+^Th1 subset, along with higher CCR3^+^Th2 and CCR5^+^Treg frequencies, in our COVID-19 cohort. The Th1 cell-polarized response is activated by the destruction of airway tissue within the airway and is parallel with previous observations in SARS-CoV and MERS-CoV infections [19]. Moreover, CD4+ cells specific for the SARS-CoV-2 spike protein have been identified in acute infection and have a Th1 cell cytokine profile [20]. Although only scarce evidence is available on other Th cell subsets in COVID-19 cases to date, higher blood plasma levels of anti-inflammatory IL-6 and IL-10 related to Th2 subsets [21,22], and lower Treg [23], were reported to be associated with patients requiring intensive care in the hospital. We observed an interesting dynamic of Th1 and Treg cell frequency in our longer-VRS cases. Th1 showed an apparent increase during the active disease and decline preceding negative conversion of PCR testing. In contrast, Treg showed time-dependent decline along the disease course. This may point out to resolving inflammation and viral clearance.

Lower NK cell counts have been correlated to disease severity [23,24], explaining the apparent decline as the disease progressed in our prolonged patients. However, why the NK cell frequencies, dominated by the circulating CD56d-NK, were also low compared to HCs during the recovery phase of our shorter VRS cases remains unknown. The upregulation of the CD8^+^CD56^+^-NKT subset suggested a possible role in antiviral mechanisms both by its direct cytolytic effect and indirect activation of antibody-producing B cells [25,26]. Along the disease course, both NKT and NKT CD8 cells showed a further increase, followed by a noticeable decline, indicating possible viral clearance and disease resolution.

In some patients, dysfunctional immune response triggers a cytokine storm that mediates widespread lung damage. A previous study identified human monocytes as the primary source of IL-1, IL-6, and nitric oxide—the main hallmarks of the event [27]. Conflicting observations on monocyte profile upon COVID-19 infection are noted. Zhang et al. found significantly increased circulating CD14^+^CD16^+^ monocytes from COVID-19 patients, with high enrichment of intermediate and non-classical subtypes [28]. However, the study did not compare the cohort with a healthy control group. On the other hand, Sanchez-Cerrillo et al. pointed out a substantial decrease in circulating monocytes in COVID-19 patients, with specific enrichment of intermediate and non-classical monocytes in the lungs of patients with the severe and critical disease [29]. Moreover, a sudden decrease in monocyte expression of HLA-DR, indicating monocyte dysfunction, immediately preceded progression to severe respiratory failure [30]. Overall, circulating CD14^+^CD16^+^ monocyte frequency was notably lower in our most prolonged VRS period patient. The CD14^+^CD16^++^-non-classical monocyte subset showed marked increase in both patients with prolonged VRS, while CD14^+^CD16^++^-intermediate monocytes were downregulated in all cohort except the patient with the longest VRS, suggesting correlation to immunopathology. A previous study has reported similar observations in uncomplicated patients compared to HCs [16].

This study has several limitations. First, only a small number of patients were enrolled in the study representing a distinct VRS period, and this may not universally reflect COVID-19 patients. Second, one of our longer VRS patients was notably older than the others, which could have impacted the immune profile and response to COVID-19 infection (reviewed in [31]). It is also worth noting that, in the present study, VRS was detected with PCR assay only, instead of virus isolation. As PCR may detect viable and non-viable viruses, further studies may adapt convincible approaches to determine the viral shedding state.

## 5. Conclusions

In the present study, we provided novel contributions to understanding the spectrum and kinetics of immune responses on uncomplicated COVID-19 patients with distinct positive VRS periods. We observed that patients with a prolonged VRS period showed an immune profile comparable to those with severe disease, as observed by lower CD4, CD8, and NK cell frequencies, although they remained clinically stable throughout hospitalization. We also characterized a dynamic of the CXCR3^+^ Th1 cell proportion, which gradually increased during the acute phase and decreased preceding viral RNA clearance, as well as anti-inflammatory CCR3^+^ Th2 and CCR5^+^ Treg and NKT cells, notably the NKT CD8 cells, which showed a high frequency on the acute period with subsequent decline along with disease resolution. While our data indicate that a unique inflammatory signature is associated with different viral RNA clearance status, further study should involve a larger cohort to define the value of these immune cell signatures as predictive biomarkers.

## Figures and Tables

**Table 1:** Clinical characteristics of patients.

Patient	01	02	03	04	Healthy Control (*n* = 4)
Sex	F	M	F	M	F (*n* = 3), M (*n* = 1)
Age (year)	24	28	60	25	Mean: 43.75
White blood cell count (% lymphocyte)	4580 (33.7)	7130 (31.3)	8460 (27.6)	6580 (40.9)	Within normal range
BMI	23.57	23.88	21.78	21.28	Mean: 22.13
Positive viral RNA shedding (day)	15	19	49	67	−
Comorbidities	−	Dyslipidemia	Right breast ductal carcinoma in situ; p Stage IA; Resected	−	−
Duration of symptom (day)	7	5	0	12	−
Symptom reported	Sore throat, dyspnea	Nasal discharge, dyspnea	−	Cough, fever	−

## Data Availability

All data generated or analyzed during this study are included in this published article.
